# Joint associations of sleep duration and physical activity with functional limitations among Chinese older adults: A cross-sectional study

**DOI:** 10.1097/MD.0000000000047175

**Published:** 2026-01-23

**Authors:** Lin Zhu, Jinfu Wang

**Affiliations:** a School of Physical Education, Sichuan University of Commerce, Chengdu, Sichuan Province, P.R. China; b Department of Physical Education, South China University of Technology, Guangzhou, Guangdong Province, P.R. China.

**Keywords:** CHARLS, functional limitations, older adults, physical activity, sleep duration

## Abstract

This study aims to investigate the independent and joint associations of sleep duration and physical activity on the risk of functional limitations in older adults, providing scientific evidence for prevention and intervention strategies. This cross-sectional study utilized data from the 2020 China Health and Retirement Longitudinal Study. Sleep duration, physical activity, and functional limitations status were collected through structured questionnaires. Multivariable logistic regression models with progressive adjustments were conducted to evaluate the independent and joint associations of sleep duration and physical activity on functional limitations risk in older adults. The final model adjusted for sociodemographic characteristics (age, gender, place of residence, marital status, education level, and personal income), health behaviors (smoking status, alcohol consumption, and social participation), and health outcomes (depressive symptoms and number of chronic diseases). Additionally, sensitivity analyses were performed to assess the robustness of the results. A total of 11,941 eligible older adults was included in this study, with 47.80% male and 52.20% female. The prevalence of functional limitations was 16.02%. Multivariable logistic regression analysis indicated that, compared to adequate sleep duration (6–8 hours), both short sleep (OR = 1.523, 95% CI: 1.352–1.716, *P* <.001) and long sleep (OR = 1.160, 95% CI: 1.004–1.339, *P* = .043) were associated with an increased risk of functional limitations (*P* <.05). Compared to the high physical activity group, the no physical activity group (OR = 2.533, 95% CI: 2.202–2.915, *P* <.001) and the low physical activity group (OR = 2.187, 95% CI: 1.819–2.629, *P* <.001) had a significantly higher risk of functional limitations (*P* <.001). The joint analysis demonstrated that combining adequate sleep duration with high physical activity was strongly associated with reduced functional limitations risk in older adults. The sensitivity analysis results were consistent with the main findings, further confirming the robustness of the results. The findings suggest that maintaining adequate sleep duration combined with a high level of physical activity is associated with a significant reduction in the risk of functional limitations among older adults. These findings provide important evidence for developing strategies to address functional limitations in older adults and highlight the potential value of comprehensive lifestyle interventions in geriatric health management.

## 1. Introduction

Functional limitations, characterized by the loss or limitation of physical function that hampers individuals’ ability to perform daily activities independently, are a prevalent and severe global health issue.^[1]^ These limitations can affect basic activities like walking, dressing, and eating, as well as instrumental activities such as shopping, cooking, and financial management.^[2,3]^ The prevalence of functional limitations varies significantly across different regions: among community-dwelling older adults in China, it ranges from 7.0% to 18.3%^[4]^; among hospitalized patients, it can reach up to 73.5%.^[5]^ In the United States, 42.3% of adults aged 65 and older experience functional limitations, while in Australia, the rate ranges from 14.0% to 39.7%.^[6]^ Japan reports a prevalence between 7.9% and 23.2%,^[7]^ and Korea sees rates from 2.24% to 22.5%.^[8,9]^ Globally, the incidence of functional limitations among older adults’ ranges from 15% to 38%.^[10]^ Functional limitations not only severely impact quality of life but also pose significant societal and economic burdens,^[11]^ and are associated with multiple chronic diseases, including cardiovascular disease, diabetes, and chronic obstructive pulmonary disease.^[10,12]^ Timely diagnosis and treatment of functional limitations are crucial to reduce healthcare utilization and long-term care demands.^[13]^

Healthy lifestyle factors, such as adequate sleep duration and sufficient physical activity, are critical in preventing functional limitations. Adequate sleep is significantly associated with a reduced risk of functional limitations, while both insufficient and excessive sleep increase this risk.^[14–16]^ Regular physical activity also reduces functional limitations risk by maintaining metabolic and immune system health, thereby lowering chronic disease incidence.^[17,18]^ However, the combined effects of sleep duration and physical activity on functional limitations risk remain underexplored, limiting our understanding of comprehensive prevention strategies. This study, using data from the China Health and Retirement Longitudinal Study (CHARLS), examines the independent and joint associations of sleep duration and physical activity with functional limitations risk in older adults, aiming to provide evidence for targeted public health interventions to reduce functional limitations and promote population health.

## 2. Methods

### 2.1. Study design

This study, using data from the CHARLS, examines the independent and joint associations of sleep duration and physical activity with functional limitations risk in older adults, aiming to provide evidence for targeted public health interventions to reduce functional limitations and promote population health.

### 2.2. Data source

This study utilized data from the CHARLS,^[19]^ a large-scale interdisciplinary project led and conducted by Peking University. CHARLS aims to collect high-quality microdata representative of households and individuals aged 45 and older in China. The dataset comprehensively covers a wide range of variables, including demographic information, health status and physical measurements, family structure and economic support, healthcare utilization and insurance, employment and retirement status, economic indicators (e.g., income, consumption, and personal assets), and community characteristics.^[19]^ These data facilitate interdisciplinary research on aging-related issues. A detailed description of the CHARLS project and its research design can be found on the official website (https://charls.pku.edu.cn/). This study was approved for data use authorization from the CHARLS research team and approved by the Peking University Institutional Review Board (IRB00001052-11015).

**Figure 1.**
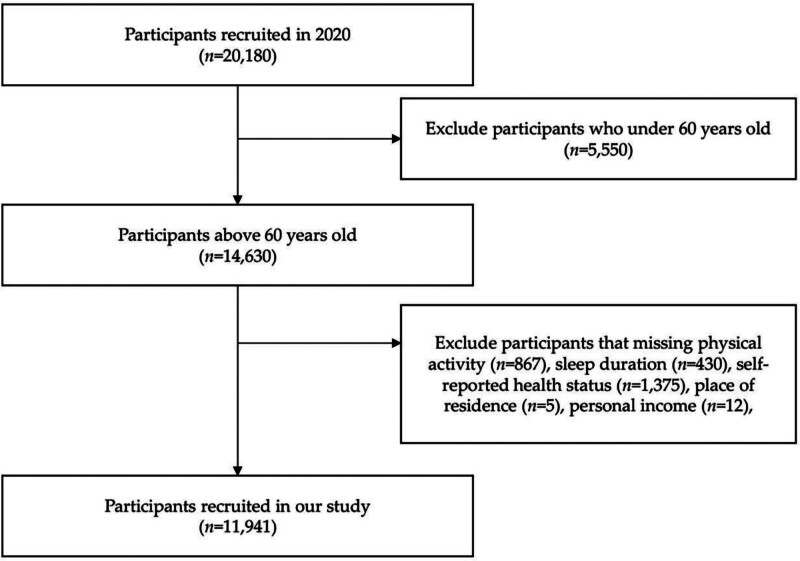
Flow of participants into study sample.

In this study, we utilized the most recent CHARLS dataset (2020), which initially included 20,180 participants. To ensure the accuracy and reliability of our analysis, we conducted rigorous screening of the sample, excluding participants who were under the age of 60 (n = 5550), and those with missing or abnormal data for key variables. Specifically, we excluded participants with missing physical activity information (n = 867), missing sleep duration information (n = 430), and missing covariate information, including self-rated health (n = 1375), place of residence (n = 5), and personal income (n = 12). Ultimately, 11,941 eligible older adults were included in the analysis. The participant selection process is illustrated in Figure 1.

### 2.3. Ethical statement

All participants provided informed consent prior to their inclusion in the study. The study protocol was reviewed and approved by the Ethical Review Committee of Peking University (approval number: IRB00001052–11,015). All procedures involving human participants adhered to the ethical standards established by the institutional and/or national research committee, and were conducted in accordance with the 1964 Helsinki Declaration and its subsequent amendments or comparable ethical standards. To ensure adherence to best practices in reporting observational studies, we have followed the STROBE checklist, which is provided in the annex.

#### 2.3.1. Measurements

##### 2.3.1.1. Sleep duration

In this study, we referred to the 2020 CHARLS questionnaire to assess participants’ average sleep duration over the past month by asking, “How many hours did you actually sleep per night on average in the past month?” and “How long was your usual nap duration in the past month?” Specifically, nap duration was quantified as follows: no napping was recorded as 0 hours; naps lasting 1 to 30 minutes were recorded as 0.5 hours; 31 to 60 minutes as 1 hour; 61 to 120 minutes as 2 hours; and naps exceeding 121 minutes as 3 hours. This approach has been adopted in previous related studies.^[20]^ Furthermore, total sleep duration was calculated by summing nighttime sleep and nap duration. Based on relevant literature, sleep duration was categorized into 3 groups: 6 to 8 hours as adequate sleep duration, <6 hours as short sleep, and more than 8 hours as long sleep.^[21]^

#### 2.3.2. Physical activity

This study assessed the physical activity levels of older adults using the short form of the International Physical Activity Questionnaire from the 2020 CHARLS dataset. The questionnaire consists of 7 items that classify physical activity levels into low, moderate, and high categories based on self-reported activity duration and weekly frequency. Since the CHARLS questionnaire provides activity duration in ranges rather than exact values, we followed previous studies^[22]^ to convert these intervals into specific time estimates: activities lasting 10 to <0.5 hours were assigned 30 minutes, 0.5 to <2 hours were assigned 60 minutes, 2 to <4 hours were assigned 180 minutes, and activities lasting 4 or more hours were assigned 240 minutes. Metabolic equivalent of task (MET) was used to quantify physical activity levels, calculated as follows: MET value = MET coefficient × weekly frequency (days/week) × daily duration (minutes/day). The assigned MET coefficients were 3.3 for low-intensity activities, 4.0 for moderate-intensity activities, and 8.0 for high-intensity activities. The total MET score was obtained by summing the values from all activity intensities. Based on prior research,^[23]^ participants were classified into 3 groups: no physical activity (0 METs/week = 1), low physical activity (1 ≤METs/week ≤599 = 2), and high physical activity (METs/week >600 = 3).

#### 2.3.3. Functional limitations

This study assessed functional limitations status in older adults using the activities of daily living (ADL) scale and the instrumental ADL (IADL) scale. The CHARLS dataset provides detailed records of 6 ADL items (dressing, eating, bathing, getting in and out of bed, toileting, and continence) and 5 IADL items (housework, cooking, shopping, medication management, and financial management). Each of these 11 items was rated on a standardized scale: no difficulty, difficulty but able to complete independently, difficulty and requiring assistance, and unable to complete. Based on established criteria for defining functional limitations and their severity,^[24,25]^ participants were classified as not disabled if they reported either “no difficulty” or “difficulty but able to complete independently” for all ADL and IADL items. Conversely, those who reported at least 1 item as “difficulty and requiring assistance” or “unable to complete” were classified as disabled. In this study, the internal consistency of the functional limitation’s measure was good, with a Cronbach α coefficient of 0.857.

#### 2.3.4. Control variables

In this study, we accounted for potential confounding factors related to sleep duration, physical activity, and functional limitations outcomes among older adults. Based on existing research,^[24,26]^ we controlled for sociodemographic characteristics, health behaviors, and health outcome factors that may influence sleep duration, physical activity, and functional limitations. The sociodemographic variables included age (1 = 60-69, 2 = 70-79, 3=≥80), gender (1 = male, 2 = female), place of residence (1 = urban, 2 = rural), marital status (1 = married, 2 = unmarried), educational level (1 = primary school or below, 2 = junior high school, 3 = senior high school and above), and personal income (1 = with income, 2 = without income). Health behavior-related variables included smoking status (1 = smoker, 2 = never smoked), alcohol consumption (1 = drinker, 2 = nondrinker), and social participation. Health outcome variables included depression status (1 = no depression, 2 = depression) and the number of chronic diseases (1 = no chronic disease, 2 = 1 chronic disease, 3 = 2 or more chronic diseases).

#### 2.3.5. Data analysis

This study used frequencies (percentages) to describe categorical variables, providing a comprehensive presentation of the sample’s overall characteristics. Chi-square tests were conducted to analyze the differences in categorical variables between groups. To explore the joint associations of sleep duration and physical activity, older adults were categorized into 9 groups: no physical activity and short sleep, no physical activity and adequate sleep, no physical activity and long sleep, low physical activity and short sleep, low physical activity and adequate sleep, low physical activity and long sleep, high physical activity and short sleep, high physical activity and adequate sleep, and high physical activity and long sleep. Multivariable logistic regression analysis was employed to examine the relationship between sleep duration, physical activity, their joint associations, and functional limitations among older adults, and odds ratios with 95% confidence intervals (CI) were calculated. Three models were reported: a crude model and 2 multivariable-adjusted models. Specifically, model 1 was unadjusted; model 2 adjusted for age and gender; model 3 further adjusted for residence, marital status, education level, household registration, personal income, smoking status, alcohol consumption, number of chronic diseases, and depression status.

To ensure the robustness of the findings, 2 sensitivity analyses were performed. First, additional relevant variables were included on top of those in the adjusted models to comprehensively exclude potential confounding factors. These variables included self-reported health status (1 = healthy, 2 = unhealthy), cognitive status (1 = cognitively normal, 2 = cognitive impairment), and pain status (1 = no pain, 2 = pain), selected based on prior studies exploring their potential impact on sleep duration, physical activity, or functional limitations.^[27,28]^ Second, we excluded older adults with extreme sleep durations (<4 or >12 hours) to further test the robustness of our results. Statistical analyses were performed using IBM-SPSS version 22.0, with a 2-tailed *P*-value of <.05 set as the threshold for statistical significance.

## 3. Results

### Participant characteristics

As shown in Table 1, a total of 11,941 participants were included in this study, with 47.80% male and 52.20% female. Among these participants, 1913 were diagnosed with functional limitations. The prevalence of functional limitations was 16.02%. Disabled individuals were typically older, had a higher proportion of females, were more likely to be married, nonsmokers, nondrinkers, lived in rural areas, had no income, and had lower educational levels. In addition, a higher proportion of disabled individuals reported multiple chronic diseases, shorter sleep durations, and depressive symptoms, although their physical activity levels were relatively high. Further analysis revealed that the distribution of functional limitations among older adults was significantly associated with gender, age, education level, type of residence, marital status, alcohol consumption, sleep duration, and physical activity levels (*P* <.001).

**Table 1:** Functional limitations status among older adults with different characteristics.

Variables	Total (N = 11941)	No-limit (N = 10028)	Limited (N = 1913)	χ^2^ values	*P*-value
Gender, n (%)					
Male	5708 (47.80)	5022 (50.08)	686 (35.86)	χ² = 130.19	<.001
Female	6233 (52.20)	5006 (49.92)	1227 (64.14)		
Age, n (%)					
60–69	5771 (48.33)	5114 (51.00)	657 (34.34)	χ² = 328.54	<.001
70–79	4559 (38.18)	3786 (37.75)	773 (40.41)		
≥80	1611 (13.49)	1128 (11.25)	483 (25.25)		
Education level					
Primary school or below	8033 (67.27)	6486 (64.68)	1547 (80.87)	χ²=200.60	<.001
Junior high school	2345 (19.64)	2091 (20.85)	254 (13.28)		
Senior high school and above	1563 (13.09)	1451 (14.47)	112 (5.85)		
Marital status, n (%)					
Married	9651 (80.82)	8219 (81.96)	1432 (74.86)	χ²=52.31	<.001
Unmarried	2290 (19.18)	1809 (18.04)	481 (25.14)		
Smoking status, n (%)					
Smoker	4804 (40.23)	4130 (41.18)	674 (35.23)	χ²=23.67	<.001
Never smoker	7137 (59.77)	5898 (58.82)	1239 (64.77)		
Alcohol consumption, n (%)					
Drinker	4093 (34.28)	3644 (36.34)	449 (23.47)	χ²=118.07	<.001
Nondrinker	7848 (65.72)	6384 (63.66)	1464 (76.53)		
Residence, n (%)					
Urban	4254 (35.63)	3721 (37.11)	533 (27.86)	χ²=59.86	<.001
Rural	7687 (64.37)	6307 (62.89)	1380 (72.14)		
Income, n (%)					
With income	2168 (18.16)	2040 (20.34)	128 (6.69)	χ²=201.50	<.001
Without income	9773 (81.84)	7988 (79.66)	1785 (93.31)		
Chronic diseases, n (%)					
No chronic diseases	1793 (15.02)	1699 (16.94)	94 (4.91)	χ²=367.80	<.001
1 Chronic disease	2532 (21.20)	2296 (22.90)	236 (12.34)		
At least 2 chronic diseases	7616 (63.78)	6033 (60.16)	1583 (82.75)		
Depression status					
Depressed	6526 (54.65)	6004 (59.87)	522 (27.29)	χ²=688.29	<.001
Not depressed	5415 (45.35)	4024 (40.13)	1391 (72.71)		
Sleep duration					
<6 h	3496 (29.28)	2665 (26.58)	831 (43.44)	χ²=228.31	<.001
6–8 h	5899 (49.40)	5186 (51.72)	713 (37.27)		
>8 h	2546 (21.32)	2177 (21.71)	369 (19.29)		
Physical activity level					
No physical activity	1376 (11.52)	939 (9.36)	437 (22.84)	χ²=404.43	<.001
Low physical activity	750 (6.28)	540 (5.38)	210 (10.98)		
High physical activity	9815 (82.20)	8549 (85.25)	1266 (66.18)		

χ² = Chi-square test.

### Relationship between sleep duration, physical activity, and functional limitations

We performed a multivariate logistic regression analysis, using functional limitations status as the dependent variable, sleep duration and physical activity level as the independent variables, and individuals with adequate sleep duration (6–8 hours) and high physical activity as the reference group. After adjusting for potential confounders (Model 3), the results indicated that compared to participants with 6 to 8 hours of sleep, those with <6 hours (OR = 1.523, 95% CI: 1.352–1.716, *P* <.001) and more than 8 hours (OR = 1.160, 95% CI: 1.004–1.339, *P* = .043) of sleep had a 1.523-fold and 1.160-fold increased risk of functional limitations, respectively, both statistically significant (*P* <.05). In terms of physical activity levels, the no activity group (OR = 2.533, 95% CI: 2.202–2.915, *P* <.001) and low activity group (OR = 2.187, 95% CI: 1.819–2.629, *P* <.001) had a 2.533-fold and 2.187-fold increased risk of functional limitations compared to the high activity group, both statistically significant (*P* <.001). Detailed data are presented in Table 2.

**Table 2:** Association between sleep duration, physical activity, and functional limitations risk among older adults in CHARLS 2020.

	Model 1		Model 2		Model 3	
	OR (95% CI)	*P*-value	OR (95% CI)	*P*-value	OR (95% CI)	*P*-value
Sleep duration						
6–8 h	1.000 (ref)	<.001	1.000 (ref)	<.001	1.000 (ref)	<.001
<6 h	2.268 (2.031–2.533)	<.001	1.988 (1.775–2.227)	<.001	1.523 (1.352–1.716)	<.001
>8 h	1.233 (1.077–1.411)	.002	1.185 (1.033–1.360)	.016	1.160 (1.004–1.339)	.043
Physical activity level						
High physical activity	1.000 (ref)	<.001	1.000 (ref)	<.001	1.000 (ref)	<.001
No physical activity	3.143 (2.765–3.572)	<.001	2.724 (2.387–3.108)	<.001	2.533 (2.202–2.915)	<.001
Low physical activity	2.626 (2.216–3.113)	<.001	2.400 (2.016–2.857)	<.001	2.187 (1.819–2.629)	<.001

Model 1 is unadjusted; Model 2 is adjusted for age and gender; Model 3 is adjusted for education level, marital status, residence, income, smoking, alcohol consumption, chronic diseases, and depression.

CHARLS = China health and retirement longitudinal study, CI = confidence interval, OR = odds ratio.

### Joint associations of sleep duration and physical activity with functional limitations

**Figure 2.**
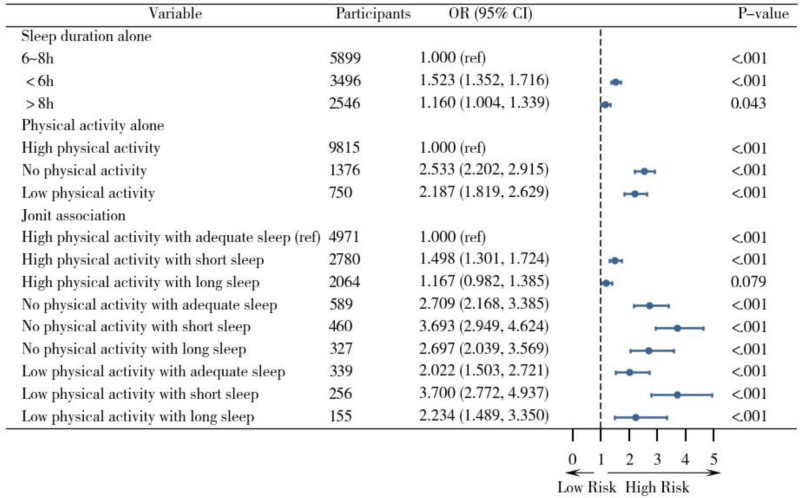
Forest plot of the independent and joint associations of sleep duration and physical activity on functional limitations. Note: This model adjusted for age, gender, education level, marital status, residence, income, smoking, alcohol consumption, chronic diseases, and depression.

Functional limitations status was the dependent variable, with sleep duration and physical activity level as independent variables. We used individuals with 6 to 8 hours of sleep and high physical activity as the reference group in a multivariate logistic regression analysis. After adjusting for potential confounders, the results showed that compared to the group with adequate sleep and moderate-to-high physical activity, the functional limitations risk was significantly higher in the following groups: short sleep with high physical activity (OR = 1.498, 95% CI: 1.301–1.724, *P* <.001), adequate sleep with no physical activity (OR = 2.709, 95% CI: 2.168–3.385, *P* <.001), short sleep with no physical activity (OR = 3.693, 95% CI: 2.949–4.624, *P* <.001), long sleep with no physical activity (OR = 2.697, 95% CI: 2.039–3.569, *P* <.001), adequate sleep with low physical activity (OR = 2.022, 95% CI: 1.503–2.721), short sleep with low physical activity (OR = 3.700, 95% CI: 2.772–4.937, *P* <.001), and long sleep with low physical activity (OR = 2.234, 95% CI: 1.489–3.350, *P* <.001), all statistically significant (*P* <.001). However, no significant association was observed in the long sleep with high physical activity group (OR = 1.167, 95% CI: 1.178–1.540, *P* = .079). See Figure 2.

### Sensitivity analyses

**Figure 3.**
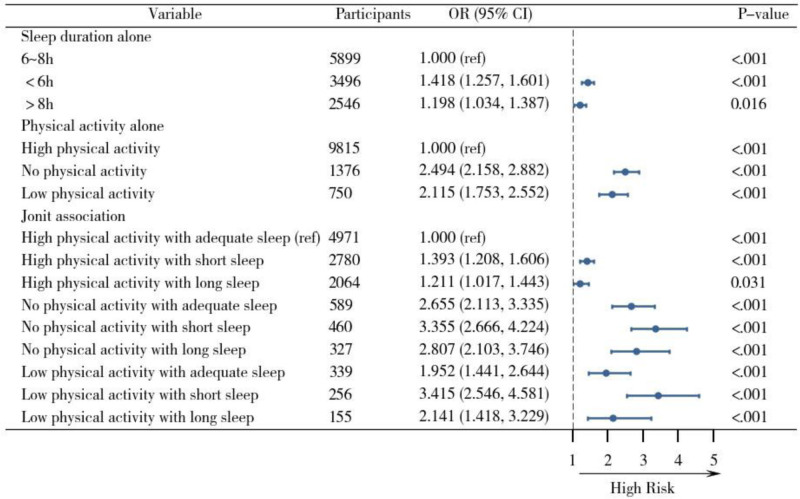
Forest plot of the independent and joint associations of sleep duration and physical activity on functional limitations, controlling for additional covariates. Note: This model adjusted for age, gender, education level, marital status, residence, income, smoking, alcohol consumption, chronic diseases, depression, self-rated health, cognitive status, and pain status.

**Figure 4.**
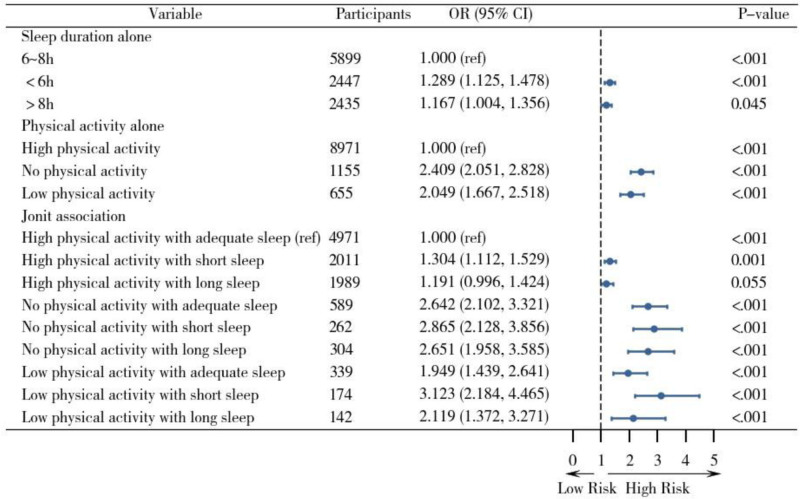
Forest plot of the independent and combined effects of sleep duration and physical activity on functional limitations, excluding sleep durations <4 or >2 h. Note: This model adjusted for age, gender, education level, marital status, residence, income, smoking, alcohol consumption, chronic diseases, and depression.

To ensure the robustness of the results, we conducted sensitivity analyses. Specifically, the main findings remained consistent even after including self-reported health, cognitive status, and pain, or excluding extreme values. This indicates that the associations observed in the initial analysis are robust (see Tables 3 and 4, Figs 3 and 4).

**Table 3:** Sensitivity analysis with additional control for self-rated health, cognitive status, and pain status.

	Model 1		Model 2		Model 3	
	OR (95% CI)	*P*-value	OR (95% CI)	*P*-value	OR (95% CI)	*P*-value
Sleep duration						
6–8 h	1.000 (ref)	<.001	1.000 (ref)	<.001	1.000 (ref)	<.001
<6 h	2.268 (2.031–2.533)	<.001	1.988 (1.775–2.227)	<.001	1.418 (1.257–1.601)	<.001
>8 h	1.233 (1.077–1.411)	.002	1.185 (1.033–1.360)	.012	1.198 (1.034–1.387)	.016
Physical activity level						
High physical activity	1.000 (ref)	<.001	1.000 (ref)	<.001	1.000 (ref)	<.001
No physical activity	3.143 (2.765–3.572)	<.001	2.724 (2.387–3.108)	<.001	2.494 (2.158–2.882)	<.001
Low physical activity	2.626 (2.216–3.113)	<.001	2.400 (2.016–2.857)	<.001	2.115 (1.753–2.552)	<.001

Model 1 is unadjusted; Model 2 is adjusted for age and gender; and Model 3 is adjusted for education level, marital status, residence, income, smoking, alcohol consumption, chronic diseases, depression, self-rated health, cognitive status, and pain status.

CI = confidence interval, OR = odds ratio.

**Table 4:** Sensitivity analysis excluding extreme sleep durations (<4 or >12 h).

	Model 1		Model 2		Model 3	
	OR (95% CI)	*P*-value	OR (95% CI)	*P*-value	OR (95% CI)	*P*-value
Sleep duration						
6–8 h	1.000 (ref)	<.001	1.000 (ref)	<.001	1.000 (ref)	<.001
<6 h	1.849 (1.630–2.097)	<.001	1.696 (1.491–1.928)	<.001	1.289 (1.125–1.478)	<.001
>8 h	1.168 (1.017–1.343)	.028	1.135 (0.985–1.308)	.081	1.167 (1.004–1.356)	.045
Physical activity level						
High physical activity	1.000 (ref)	<.001	1.000 (ref)	<.001	1.000 (ref)	<.001
No physical activity	2.966 (2.571–3.423)	<.001	2.606 (2.249–3.020)	<.001	2.409 (2.051–2.828)	<.001
Low physical activity	2.529 (2.096–3.052)	<.001	2.308 (1.905–2.797)	<.001	2.049 (1.667–2.518)	<.001

Model 1 is unadjusted; Model 2 is adjusted for age and gender; and Model 3 is adjusted for education level, marital status, residence, income, smoking, alcohol consumption, chronic diseases, depression, self-rated health, cognitive status, and pain status.

CI = confidence interval, OR = odds ratio.

## 4. Discussion

This study systematically explored the independent and combined effects of physical activity and sleep duration on the risk of functional limitations among older adults using the CHARLS 2020 data. The results revealed that both high levels of physical activity and adequate sleep duration were significantly associated with a reduced risk of functional limitations. Moreover, the combination of high physical activity and adequate sleep duration is associated with a more pronounced reduction in functional limitations risk. Sensitivity analyses further confirmed the robustness of these findings, highlighting the important role of physical activity and sleep duration in preventing functional limitations.

The relationship between sleep duration and functional limitations risk has been widely explored in numerous studies. For example, Peng et al found that both short and long sleep durations were associated with a higher likelihood of difficulties in daily activities.^[14]^ Similarly, Seixas et al reported that individuals with longer sleep durations were more likely to experience functional limitations compared to those with shorter sleep durations.^[29]^ Furthermore, Zhang et al also pointed out that older adults with shorter sleep durations had a higher risk of functional limitations.^[30]^ However, this study did not find a significant association between long sleep duration and functional limitations, possibly due to the limited sample size (n = 1859) and potential detection bias. In contrast, a study from the United States indicated that individuals with longer sleep durations were more likely to encounter daily activity difficulties compared to those with average sleep durations. Our findings align with studies emphasizing that both short and long sleep durations increase functional limitations risk,^[31]^ further supporting the importance of adequate sleep duration in preventing functional limitations. Adequate sleep duration is often closely linked to physical health and may reduce the risk of functional limitations through various mechanisms. First, adequate sleep helps maintain normal body functions, as insufficient or excessive sleep is a major risk factor for functional decline. A healthy sleep pattern helps older adults stay energized, reduce fatigue accumulation, and lower the risk of functional impairment in various body systems.^[32]^ Additionally, adequate sleep duration is closely related to metabolic function, supporting the immune system, improving overall metabolic health, and reducing the risk of chronic diseases. Numerous studies have shown a strong correlation between chronic diseases and the development of physical functional limitations,^[33]^ and adequate sleep can mitigate this association by promoting better health. On the other hand, short sleep duration may disrupt these physiological processes, affecting cognitive function and physical recovery capabilities, thereby increasing the risk of functional decline.^[34]^ Furthermore, long sleep duration may be associated with chronic inflammation, insulin resistance, and cardiovascular health issues, all of which may contribute to functional decline and functional limitations.^[35,36]^

This study explored the relationship between physical activity and functional limitations risk among older adults. The results indicated that physical activity is significantly associated with a reduced risk of functional limitations. These findings align with existing literature. A 9-year prospective study found that active older adults had a lower risk of functional limitations compared to inactive individuals (Hazard ratio = 0.48, 95% CI: 0.25–0.94).^[37]^ Another study based on the Chinese CHARLS data revealed that older adults with higher physical activity levels had significantly lower risks of frailty and functional limitations.^[38]^ Additionally, a prospective cohort study found that regular vigorous physical exercise reduced the risk of functional limitations-related retirement,^[39]^ consistent with our findings. However, Lahti et al observed that moderate exercise did not reduce functional limitations risk among women, while sufficient vigorous activity significantly lowered the risk of functional limitations-related retirement (OR = 0.43, 95% CI: 0.29–0.64).^[40]^ This discrepancy may be attributed to differences in how physical activity was measured and individual characteristics. Different studies use varying standards to define and measure vigorous and moderate activities. Some studies quantify activity based on duration, heart rate changes, or energy expenditure, while others rely on self-reported data. These varying methods can affect the accuracy and objectivity of the data. Moreover, individual characteristics such as age, health status, and baseline functional abilities vary across older adults, influencing both activity assessments and outcomes. In this study, physical activity levels were categorized into 3 groups based on MET-min/week: no physical activity (0 MET-min/week, 23.42%), low-level physical activity (1–599 MET-min/week, 12.73%), and high-level physical activity (≥600 MET-min/week, 63.85%). This classification follows the World Health Organization’s recommendation that adults engage in at least 150 minutes of moderate-intensity or 75 minutes of vigorous-intensity activity per week, equating to 600 MET-min/week.^[41]^ Based on this, this study defined ≥ 600 MET-min/week as high-level physical activity to more accurately reflect older adults’ activity levels. Therefore, differences in how physical activity is defined and measured across studies may lead to varying conclusions. The prevention of functional limitations through physical activity may occur through several mechanisms. First, physical activity helps maintain and enhance basic functional abilities,^[42]^ such as muscle strength, balance, and walking speed, which are critical in preventing functional limitations. For example, a study found that older adults unable to complete 5 repeated sit-to-stand tests had a significantly higher risk of functional limitations.^[43]^ Second, physical activity improves cardiovascular health and reduces the risk of chronic diseases, such as hypertension and diabetes, which are common causes of functional limitations.^[44]^ Additionally, physical activity can enhance mental health by reducing depression and anxiety.^[45]^ Previous studies have confirmed that increased emotional distress, including depression, predicts the deterioration of physical function within 4 years, with varying levels of depression influencing functional limitations risk among older adults.^[46]^ Thus, engaging in physical activity may reduce the incidence of functional limitations by improving mental health and enhancing overall well-being. While this study, along with previous literature, provides valuable insights, further research is needed to fully understand the mechanisms linking physical activity to functional limitations. This will help develop more effective prevention and treatment strategies to improve the quality of life for disabled older adults.

This study is one of the few to reveal the joint associations of adequate sleep duration and sufficient physical activity with a reduced risk of functional limitations among older adults in China. Specifically, combining an adequate sleep duration (e.g., 7 hours per night) with high levels of physical activity appears to significantly reduce the incidence of functional limitations. This finding suggests that interventions focusing solely on sleep or exercise may not fully address the issue of functional limitations in older adults, and their combined effect may be crucial. Additionally, related studies have observed the positive impact of the interaction between sleep duration and physical activity on other health conditions. For example, Liu et al found that, compared to low levels of physical activity and long sleep duration, adequate sleep duration and high levels of physical activity can significantly reduce cognitive decline associated with aging.^[47]^ Another study demonstrated that combining normal sleep duration (6–8 hours/day) with physical activity can significantly reduce the risk of type 2 diabetes (OR = 0.65, 95% CI: 0.46–0.91).^[48]^ Cheng et al found that regardless of sleep duration, participants who met the World Health Organization’s physical activity recommendations had a lower likelihood of developing hypertension compared to those who did not meet the recommended activity levels.^[49]^ Overall, this study suggests that combining adequate sleep duration with sufficient physical activity offers potential benefits in reducing functional limitations risk. Future research should further explore the synergistic mechanisms of these factors to provide more comprehensive intervention strategies for preventing functional limitations.

### 4.1. Strengths and limitations

This study has several strengths. First, it extends previous research by exploring the synergistic relationship between sleep duration, physical activity, and functional limitations, providing new insights into their interplay in older adults. Second, the study is based on CHARLS, a nationally representative prospective cohort, using the latest 2020 CHARLS data, ensuring both timeliness and the broad applicability of the results. The comprehensive data provided by CHARLS enabled an in-depth analysis of the relationship between sleep duration, physical activity, and functional limitations risk, while controlling for multiple potential confounders. Furthermore, we conducted a series of sensitivity analyses to ensure the robustness of the results. However, there are some limitations that should be noted. First, the assessment of sleep duration and physical activity was based on self-reported data, which may introduce recall bias or reporting errors.^[50]^ Although these variables have been validated in previous studies,^[20]^ future research should consider using objective measurements, such as wearable devices and polysomnography, to reduce such biases.^[51,52]^ Second, functional limitations were also self-reported, not clinically diagnosed, which may lead to an underestimation or overestimation of the prevalence of functional limitations. Future studies should employ objective assessments to more comprehensively and accurately evaluate the prevalence of functional limitations and their impact on health. Third, the cross-sectional design of this study limits the ability to establish causal relationships between sleep duration, physical activity, and functional limitations and does not allow for the exclusion of reverse causality, where functional limitations may influence sleep or physical activity patterns. Future longitudinal studies are needed to confirm these findings and strengthen the evidence for the role of sleep duration and physical activity in the prevention and management of functional limitations. Additionally, we used the binary classification method to define the status of functional limitations (presence or absence). While this method is widely used in similar studies and can effectively evaluate overall risk, it ignores the ordinal nature of functional limitations. In other words, this method cannot distinguish between different levels of functional limitations, such as mild, moderate, and severe. This simplification may lead to information loss and reduce the sensitivity of the analysis. Future studies can consider adopting more complex modeling strategies, such as ordinal logistic regression or multinomial logistic regression, to better preserve the ordinal nature of the data and explore the different dimensions of functional limitations.^[2]^ Lastly, although we have accounted for several potential confounders in our analysis, other relevant variables, such as BMI, diet, household income, and medication use, may still have influenced the results. Future studies should consider including these variables to provide a more comprehensive understanding of the relationships between sleep duration, physical activity, and functional limitations in older adults.

## 5. Conclusion

This study, using a cross-sectional design, highlights the significant protective role of adequate sleep duration and high levels of physical activity in reducing the risk of functional limitations among older adults. The results further suggest that the synergistic effect of sleep duration and physical activity has a significant impact on preventing functional limitations in this population. Therefore, integrated lifestyle interventions that optimize sleep duration and increase physical activity are crucial for improving older adults’ health and preventing functional limitations. This study emphasizes the importance of balancing sleep and physical activity in daily life to promote better health outcomes.

## Acknowledgments

The authors are grateful to the CHARLS researchers and the CHINA Data Service for making the CHARLS data available online freely. We also would like to thank all the survey participants who provided these data.

## Author contributions

**Conceptualization:** Jinfu Wang.

**Data curation:** Lin Zhu.

**Formal analysis:** Lin Zhu, Jinfu Wang.

**Methodology:** Lin Zhu, Jinfu Wang.

**Writing – original draft:** Lin Zhu.

**Writing – review & editing:** Jinfu Wang.
